# Supplementary material for: Smooth Interpolating Curves with Local Control and Monotone Alternating Curvature
Source: Comput Graph Forum. 2022 Oct 6;41(5):25–38. doi: 10.1111/cgf.14600 (PMC9827861; doi:10.1111/cgf.14600)
Supplement: Supplementary file 1 — Supplement Material [file CGF-41-25-s001.zip › Local-Smooth-Interpolating-MonoCurvature/extern/clothoids/submodules/Utils/docs/api-cpp/program_listing_file_Utils_CPU_info.hh.html]

Program Listing for File Utils\_CPU\_info.hh — Utils v1.2

### Navigation

- index
- toc
- Utils »
- Program Listing for File Utils\_CPU\_info.hh

# Program Listing for File Utils\_CPU\_info.hh¶

↰ Return to documentation for file (`Utils_CPU_info.hh`)

```
/*--------------------------------------------------------------------------*\
 |                                                                          |
 |  Copyright (C) 2011                                                      |
 |                                                                          |
 |         , __                 , __                                        |
 |        /|/  \               /|/  \                                       |
 |         | __/ _   ,_         | __/ _   ,_                                |
 |         |   \|/  /  |  |   | |   \|/  /  |  |   |                        |
 |         |(__/|__/   |_/ \_/|/|(__/|__/   |_/ \_/|/                       |
 |                           /|                   /|                        |
 |                           \|                   \|                        |
 |                                                                          |
 |      Enrico Bertolazzi                                                   |
 |      Dipartimento di Ingegneria Industriale                              |
 |      Universita` degli Studi di Trento                                   |
 |      email: enrico.bertolazzi@unitn.it                                   |
 |                                                                          |
\*--------------------------------------------------------------------------*/

#ifndef UTILS_CPU_INFO_HH
#define UTILS_CPU_INFO_HH

#include <string>

namespace Utils {

  #ifndef DOXYGEN_SHOULD_SKIP_THIS
  using std::string;
  void cpuId( unsigned long CPUInfo[4], unsigned long l );
  #endif

  void
  info(
    bool & bMMX,
    bool & bMMXplus,
    bool & bSSE,
    bool & bSSE2,
    bool & bSSE3,
    bool & bSSSE3,
    bool & bSSE41,
    bool & bSSE42,
    bool & bSSE4a,
    bool & bSSE5,
    bool & b3Dnow,
    bool & b3DnowExt
  );

  bool has_MMX();
  bool has_MMXplus();
  bool has_SSE();
  bool has_SSE2();
  bool has_SSE3();
  bool has_SSSE3();
  bool has_SSE41();
  bool has_SSE42();
  bool has_SSE4a();
  bool has_SSE5();
  bool has_3Dnow();
  bool has_3DnowExt();

  string cpuInfo();
}

#endif
```

### Quick search

### Table of Contents

- rang - Colors for your Terminal
- Zstr
- C++ API

«
hide menu

menu
sidebar
»

### Navigation

- index
- toc
- Utils »
- Program Listing for File Utils\_CPU\_info.hh

© Copyright 2021, Enrico Bertolazzi.
Created using Sphinx 4.2.0.
